# Why sex and gender matter in implementation research

**DOI:** 10.1186/s12874-016-0247-7

**Published:** 2016-10-27

**Authors:** Cara Tannenbaum, Lorraine Greaves, Ian D. Graham

**Affiliations:** 1 Insitute of Gender and Health, Canadian Institutes of Health Research, Ottawa, Canada; 2 Université de Montréal, Montréal, Canada; 3 British Columbia Centre of Excellence for Women’s Health, Vancouver, Canada; 4 University of Ottawa, Ottawa, Canada

**Keywords:** Sex, Gender, Knowledge translation, Implementation science, Realist evaluation, Gender transformative approaches

## Abstract

**Background:**

There has been a recent swell in activity by health research funding organizations and science journal editors to increase uptake of sex and gender considerations in study design, conduct and reporting in order to ensure that research results apply to everyone. However, examination of the implementation research literature reveals that attention to sex and gender has not yet infiltrated research methods in this field.

**Discussion:**

The rationale for routinely considering sex and gender in implementation research is multifold. Sex and gender are important in decision-making, communication, stakeholder engagement and preferences for the uptake of interventions. Gender roles, gender identity, gender relations, and institutionalized gender influence the way in which an implementation strategy works, for whom, under what circumstances and why. There is emerging evidence that programme theories may operate differently within and across sexes, genders and other intersectional characteristics under various circumstances. Furthermore, without proper study, implementation strategies may inadvertently exploit or ignore, rather than transform thinking about sex and gender-related factors. Techniques are described for measuring and analyzing sex and gender in implementation research using both quantitative and qualitative methods.

**Summary:**

The present paper describes the application of methods for integrating sex and gender in implementation research. Consistently asking critical questions about sex and gender will likely lead to the discovery of positive outcomes, as well as unintended consequences. The result has potential to strengthen both the practice and science of implementation, improve health outcomes and reduce gender inequities.

**Electronic supplementary material:**

The online version of this article (doi:10.1186/s12874-016-0247-7) contains supplementary material, which is available to authorized users.

## Background

Efforts to integrate sex and gender throughout all phases of the health research cycle have been rising sharply over the past two decades [1–4]. Since 2010, the Canadian Institutes of Health Research has been requiring researchers to indicate whether their research protocol accounts for sex or gender, using the term “sex” to refer to the biological attributes that distinguish male from female, and the term “gender” to refer to men and women’s socially constructed roles, identities and behaviors [5, 6]. As of 2016, the U.S. National Institutes of Health Research asks applicants to explain how they plan to factor consideration of sex as a biological variable into their research design and analysis [7, 8]. The Gender Advisory Group to the European Framework Program for Research and Innovation also mandates the Gender Dimension across all sectors [9]. Journal editors are encouraged to increase accountability around sex and gender reporting requirements, by using the Sex and Gender Equity in Research (SAGER) guidelines [10, 11]. These events beg the question: how have research methods in implementation science addressed sex and gender? For the purpose of this article, we will use the term implementation research and practice (IRP) to include knowledge translation, implementation research and practice.

The opening argument for this debate article is that to date, despite the evidence on the impact of sex and gender on health, research methods in the field of implementation have neglected sex and gender considerations. An analysis of selected literature in IRP supports this proposition. For example, a review of the tables of contents and indexes of three popular implementation science texts [12–14] reveals that none devote a chapter to the role of sex and gender in implementation science. Only one includes gender in the index [14], which refers to a chapter in the text with a few lines describing how many sexually transmitted infection interventions targeting racial/ethnic minorities are gender specific and how the strategies to reach men and women may differ [15].

Searching the top 10 articles of 2015 as reported by Implementation Science (see Additional file 1: Appendix 1) for the words sex or gender shows that only one makes a minor mention of gender as it relates to controlling for ‘clinician gender’ in a modeling exercise [16]. Sex and gender also do not appear to play a prominent role in implementation theories. For example, in Nilsen’s review [17] of implementation theories, models and frameworks, only 2 make minor references to gender [18, 19]. One simply includes ‘gender’ as one of the barriers to optimal clinical practice under the category “health care professional/physician barriers” [18] and the other includes a footnote about a study they were citing, that “a fourth factor, gender of participants, was also related to program outcomes but was not included in their subsequent analysis” [19]. Furthermore, neither of the germinal papers on the Theoretical Domains Framework [20, 21], a widely used and influential framework [22] that guides assessment of barriers to implementation, makes any reference to sex or gender. There is a domain in the framework that focuses on ‘social/professional role and identity’, which could capture elements of sex and gender, but usually tends to be limited to assessing professional roles and almost never identity, let alone sex or gender identity.

Turning to the knowledge synthesis literature on implementation, the Cochrane Collaboration’s Methods Equity group [23] is active in increasing awareness of the need and methods for sex and gender analysis in systematic reviews [23–25]. Both the Effective Practice and Organisation of Care (EPOC) and Consumers and Communication review groups have official guidance in their resources for authors on equity [26, 27]. A recent assessment of a sample of systematic reviews from these two groups however, reveals limited consideration of sex and gender in the written report. For example, of 12 EPOC and seven Consumer and Communication reviews published between July 2014 and May 2015, none addressed sex or gender in the analysis or implications sections of the report (Personal Communication, Jennifer Petkovic, Peter Tugwell and Vivian Welch on behalf of the Campbell and Cochrane Equity Methods Group, April 13, 2016). It is possible that the review authors did consider sex and gender in their analyses and determined it was unimportant. However, they failed to report this.

Little research has been undertaken or reported to inform how sex and gender impact IRP, as evidenced by this analysis of key texts, well-used conceptual models, and Cochrane reviews on implementation strategies. The objective of this paper is to describe the rationale for why and how sex and gender should be considered in IRP.

## Discussion

### What is sex? What is gender?

A first step for understanding how to integrate sex and gender in IRP involves operationalizing the two terms, and recognizing different components of gender. The term sex refers to a biological construct, whereby an individual is defined as being male or female according to genetics, anatomy and physiology [6, 7, 11, 28–32]. Researchers should use the term sex when describing the number of male or female patients or committee members, or when stratifying outcomes by male versus female participants or health care providers. It is more appropriate to say what the distribution by sex of a sample or target audience is, than to use the term ‘gender distribution.’ This is because gender is a multifaceted and fluid construct, influenced in a temporal manner by social and cultural contexts and environments to create gender norms [6, 7, 11, 28, 30–35]. Gender norms influence commonly accepted ways of how people behave, how they perceive themselves and each other, how they act and interact, and the distribution of power and resources in society [6, 28, 31–35]. Gender can be structured by, and operating within ethnicity, indigenous status, social status, sexuality, geography, socioeconomic status, education, age, disability/ability, migration status, and religion, requiring an intersectional approach to implementing practices, programs and policies [36, 37]. The acronym “PROGRESS” can be used to remember these variables: place of residence, race/ethnicity/culture/language, occupation, gender/sex, religion, education, socioeconomic status, and social capital [38]. Researchers often understand gender as a function of gender roles (e.g. child care, housework), gender identity (e.g. personality traits such as being sensitive to the needs of others or having leadership abilities), gender relations (e.g. social support), and institutionalized gender (e.g. career opportunities, personal income, educational background) [6, 28, 34]. Gender as a broad term can also refer to the expressions and identities of girls, women, boys, men, and gender diverse people [39, 40]. For this reason, definitions of sex and gender are evolving as science changes, and it remains challenging to easily separate the biological from the social. Sex and gender are often interrelated, interactive and potentially inseparable [6, 11]. Given the epistemology of knowledge, and the social nature of implementation and behavior change, the effect of gender and other identity factors, either alone or in combination, can serve as barriers or enablers to the outcome or impact of IRP interventions.

### Measuring and understanding sex and gender

Collecting and analyzing data on sex in IRP is relatively simple if using typical male and female categories. Sex can be self-reported, designated by an examination of external genitalia, or genetically determined based on an XX, XY or intersex genotype [11]. Data on sex-related factors can include measuring sex hormones, body and organ size, metabolism, or fat tissue distribution [41]. Gender is more complex, and can be operationalized along four different constructs: gender roles, gender identity, gender relations and institutionalized gender [6, 28, 31, 32]. Table 1 defines these four constructs, gives examples of key questions that can be asked of each in IRP, and lists measures and methods for use in IRP research [6, 28, 31, 32, 42–44].

**Table 1:** Relevance of four gender constructs to implementation research and practice

Gender construct	Definition	Examples of potential questions for implementation research	Examples of potential questions for implementation practice	Examples of measures
Gender roles [6, 28]	Represent the behavioral norms applied to men and women in society, which influence individuals’ everyday actions, expectations, and experiences. Gender roles often categorize and define individuals within the family, the labour force, or the educational system. May form the basis for stereotypes.	How can considering gender roles help us understand and anticipate barriers and opportunities facing health-care professionals in the uptake of new interventions?	How can considering gender roles help inform dissemination strategies that are successful in reaching different audiences where they are?	The Gender Role Conflict Index [73]
				Other variables such as occupation [45], primary breadwinner status, time doing household chores and caregiving responsibilities can also be used to capture gender roles in data collection and analysis [46].
Gender identity [6, 28, 32, 39, 40, 42]	Describes how we see ourselves, and are seen by others, as female or male, or across a feminine-masculine continuum. Individuals may also self-identify dynamically along the continuum of gender-queer and/or transgender. Gender identity affects our feelings and behaviors.	Do a range of gender identities need to be considered when asking the question, “for whom does this implementation strategy work and under which circumstances?”	Will the reach of the implementation intervention extend to male, female and transgender individuals?	The BEM sex role inventory [74]
				The Personal Attributes Questionnaire [75]
			Should the content of the implementation intervention consider gender identity or sexual orientation?	The Conformity to Masculine Norms Inventory [76],
				A two-step approach to measuring gender identity first asks individuals to indicate their sex assigned at birth (male/female), and then asks the same individuals how they currently self-identify (male, female, trans male/trans man, trans female/trans woman, gender queer/gender non-conforming) [39, 40].
Gender relations [6, 28, 33]	Refers to how we interact with or are treated by people in the world around us, based on our ascribed or experienced gender.	How might the outcomes of implementation interventions differ by sex and gender according to whether the degree to which the geographic setting is culturally homogeneous, diverse or gender equitable?	What are the implications of an implementation intervention being communicated or delivered to women only, men only, men and women separately or together? How is this mediated by cultural context?	The Self-Perceived and Self-Reported Gender Equality scale [77].
Institutionalized gender [6, 28, 33]	Reflects the distribution of power between men and women in the political, educational, and social institutions in society. The institutionalized aspect of gender also shapes social norms that define, reproduce, and often justify different expectations and opportunities for men and women.	If particular decision-maker groups value, use, or require, different kinds of knowledge, have you considered how institutionalized gender might play a role? How might this change over time?	How can dissemination messages be crafted in a way that responds to sex and gender–related factors without reproducing or exploiting any negative stereotypes embedded in institutionalized gender?	The use of qualitative methods (e.g. case studies, ethnography, narrative and descriptive qualitative approaches etc.) can be used to explore concepts of institutionalized gender, and to gain a more in-depth understanding of gender as a barrier or enabler.

Traditionally, individuals are asked to categorize their sex as male or female and many assumptions, often based in gender and not biology, are made on the basis of their responses. Researchers are now rethinking this approach to be more inclusive of gender identity and expression [39]. A two-step approach to measuring sex and gender identity could first ask individuals to indicate their sex assigned at birth (male/female), and then ask the same individuals how they currently self-identify, which could include male, female, trans male/trans man, trans female/trans woman, gender queer/gender non-conforming; and provide space to self identify as another option not provided [40]. Similarly, participants could also be given the option to disclose sexual orientation and whether they consider themselves part of the lesbian, gay, bisexual or transgender (LGBT) community.

The scales in Table 1 list measures that can be used to quantify different dimensions of gender. Researchers can also create gender scales using gender-related variables of relevance to their particular research topic [45, 46]. Pelletier et al. created a composite gender score using 7 characteristics: 1) status on primary earner; 2) personal income; 3) number of hours per week spent doing housework; 4) status of primary person responsible for doing housework; 5) level of stress at home; 6) masculine traits; and 7) feminine traits. They were able to demonstrate that gender, independent of sex, predicts poor outcome after acute coronary syndrome, pointing to new areas of intervention [44].

Qualitative methods are also useful for the collection of data on specific dimensions of gender. Case studies, ethnography, narrative and descriptive qualitative approaches can provide evidence and contextualized insight across a range of participants’ personal characteristics, including those of sex and gender. Qualitative methods can also be used to explore concepts of institutionalized gender, and to gain a more in-depth understanding of gender as a barrier or enabler to the use of implementation interventions, the uptake of the evidence-informed clinical interventions or program and the outcomes of implementation efforts. A number of texts, casebooks, examples and online courses are available that provide guidance on how to conduct sex and gender science using commonly employed quantitative and qualitative methods [6, 32, 42, 43].

### The case for considering sex and gender in implementation research methods

Emerging evidence suggests that sex and gender are important in decision-making, stakeholder engagement, communication and preferences for the uptake of interventions. Furthermore, when gender norms, identities and relations are ignored, unintended consequences may occur. The following five scenarios give examples of when and why sex and gender should be measured and considered in implementation research:

1. **When the implementation of an intervention requires decision-making on the part of individuals or organizations**. Decision-making is a critical component of behavior change interventions, and plays a key role in the uptake of new organizational practices and programs [47]. Research from the fields of business and management offer insights for IRP on important sex and gender factors related to decision-making [48–50]. Qualitative research conducted by Deloitte Consulting with 18 large business organizations suggests that female executives have a tendency to be more attuned to micro-level signaling during meetings, and may favour discovery options and iterative thinking during decision-making processes [48]. Male executives tend to end a conversation once they connect with a good idea or solution. Their female counterparts are inclined to be more inquisitive, wanting to hear everyone’s thoughts before deciding, and taking more time to find the ideal solution. Different leadership traits among male and female leaders can therefore influence the outcome of decision-making processes [49, 50].
2. **When sex and gender dynamics may play a role in stakeholder engagement and conflict resolution**. A survey queried reasoning methods among 624 corporate board directors, of whom 75 % were male and 25 % female [51]. Female directors scored significantly higher scores on the complex moral reasoning dimension, which implies attending more to relationships and to the challenge of balancing multiple stakeholders’ interests. Females may also engage in more collaboration and consensus building, not only to make sound decisions but also to elicit common support for a course of action [49, 50]. The outcome of an implementation intervention may therefore depend on the sex and gender dynamics in each particular context.
3. **When communication strategies are being tested, as sex and gender may be differentially responsive to the choice of language used, the strength of persuasion of the communication strategy, and the way promotional information is processed**. This is why sex and gender form the basis for market segmentation in the fields of marketing and consumer behaviour, where subtle changes in language and emotional appeal can have a differential effect on men and women’s attitudes towards the brand advertised and purchase intentions [52]. The way messages and interventions are primed or packaged to reflect gender norms or stereotypes may also influence the outcomes of health promotion interventions. For instance, priming individuals to the perception that women eat healthier foods than men leads both male and female study participants to prefer healthy foods, whereas priming masculinity results in unhealthy food preferences [53]. When the packaging and healthiness of the food are gender congruent (i.e., feminine packaging for a healthy food, masculine packaging for an unhealthy food) both male and female participants rate the product as more attractive, report that they would be more likely to purchase it, and even rate it as tasting better compared to when the product is stereotype incongruent.
4. **When negative or harmful gender stereotypes may impede the uptake and outcomes of an IRP initiative** [54]. A realist review of the implementation of school-based interventions to prevent domestic abuse for children and young people reported that lesbian, gay, bisexual and transgender youth felt excluded from the programmes, as the content did not address gender identity or sexual orientation in high-risk populations [55]. Similarly, data suggest that masculine norms around emotional control and self-reliance are associated with recurrent non-suicidal self-injury [56]. Stigma related to healthcare seeking for male depression and suicide [57, 58], may explain why women are more likely to benefit from psychosocial treatment for the prevention of suicide and suicidal ideation compared to men [59]. Some studies purport that gender bias in prescription patterns among health care providers results in more women receiving treatment with antidepressants for mental health [60] and pain symptoms, but only among female clinicians [61]. Men, on the other hand, may be preferentially managed with orthopaedic surgery to manage knee arthritis [62].
5. **When gendered power relations may inadvertently skew the uptake of information focused on women’s health needs, such as maternal and child health, sexual and reproductive health, or family planning** [63]. This occurs in cultures and settings where male partners and head of households play a large role in female’s health-seeking behaviour due to their authority and decision-making role. For example, the introduction of health programs enhanced by mobile phone technologies overall fosters women’s empowerment in low-income countries [64]. However, in some cases these programs exacerbated gender relations and gender inequalities, such as when women were pressured to give the phones provided by the program to their husband if he did not already own a phone, or when conflicts about phone use led to cases of spousal abuse.

**Fig. 1:**
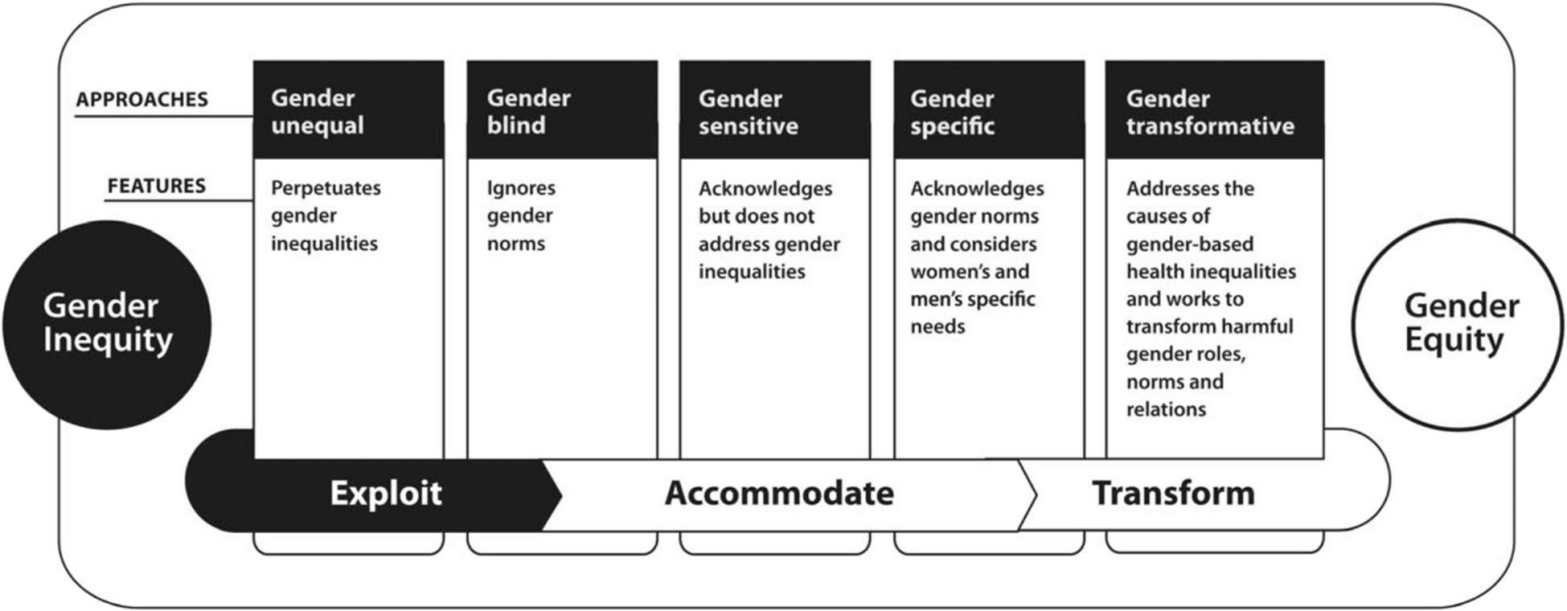
A continuum of approaches for integrating sex and gender. Reproduced with permission from: Lorraine Greaves, Ann Pederson, Nancy Poole (Eds). *Making It Better: Gender Transformative Health Promotion.* Canadian Scholar’s Press/Women’s Press. 2014. Available at http://promotinghealthinwomen.ca/wordpress/wp-content/uploads/2015/02/Continuum-of-Approaches_colour.pdf Accessed March 20, 2016

The World Health Organization outlines a spectrum of gender-responsive programs, illustrating the progression from the exploitative use of gender stereotypes in IRP messaging, through to accommodation and ultimate transformation to gender equity (Fig. 1) [65, 66]. Making active choices reflecting content, messaging and decision-making processes during the implementation of an intervention can have a critical impact on gender equity for women and men. Gender transformative approaches are preferred as they anticipate unintended barriers and consequences and address the causes of gender-based health inequities where they exist [67]. For example, during implementation of a tobacco control program, investigators can decide to use motivational recruitment techniques that appeal to a person’s health and self-respect, as opposed to messaging that invokes and reinforces stereotypical gendered norms of sexual attractiveness, beauty claims or images based on masculinity or femininity [63]. Recent guidance based on qualitative research suggests de-linking messages for men and for women when promoting tobacco reduction during pregnancy and post partum, since the uptake of the intervention can be hindered by negative couple dynamics if the partners have different smoking behaviours or attitudes about smoking during this period [68–70]. Another transformative approach to encourage uptake of smoking cessation interventions would be to focus on a wider range of non-stereotypical gendered roles that include fathering for men and work for women as potential motivators.

Sex and gender can be therefore be pivotal at multiple points along the IRP process, from the content and messaging surrounding the intervention, to decision making around the uptake and unintended consequences of an intervention. Asking sex and gender questions can also elucidate enablers and barriers to the adoption of complex behavioral interventions. For example, examining the outcome of implementing a multidisciplinary cardiac rehabilitation program merits asking whether women or men have less time to devote to recovery and prevention activities due to gender-based expectations regarding their responsibilities at home. The potential advantages of including sex and gender in the study of other complex behavioral interventions (e.g., hand hygiene, reducing clinician's opioid prescribing, reducing falls in hospital, increasing vaccination rates, and obesity prevention) require further investigation. Measuring the way sex and gender influences these interventions may help elucidate potential mechanisms and contexts behind the success or failure of various IRP efforts, as shown in the examples above on tobacco cessation, healthy eating, depression and suicide, pain, heart disease and domestic violence.

### Some research questions to drive the selection of methods

Researchers can start by asking a series of questions about how sex and gender can have an impact on their implementation initiative in order to determine the best way to measure and analyze the effect of sex and gender. First, how might sex or gender affect decision-making and stakeholder engagement, or facilitate or impede the uptake of evidence-informed practice, programs, policies? Second, how might sex based characteristics or prevailing gender norms or gender roles serve as barriers or enablers to the uptake of evidence-informed practices, programs, policies? Third, when and how should the communication strategy, wording or messaging be tailored across sex, gender or other identity characteristics? Fourth, when using participatory/collaborative or integrated knowledge translation research approaches, could the sex and gender of the researchers and knowledge users matter, and if so, how? Similarly, how might gender relations as a function of dyads or interpersonal dynamics within an organization, community, workplace or institution influence the outcome of the intervention? And finally, should the research protocol consider examining whether there are unintended impacts of implementation that exacerbate or diminish sex, gender or other diversity-related inequities? Table 2 lists a series of questions for researchers to consider when designing their IRP research. Additional opportunities for integrating sex and gender in IRP as relates to models of health systems research have been reviewed in detail elsewhere [33].

**Table 2:** Some sex and gender research questions for researchers studying implementation

• Are theories of behaviour change (i.e. processes of reasoning or reflection) equally applicable across sexes, genders and other intersecting variables?
• How does consideration of sex, gender and diversity affect the assessing of barriers and supports to uptake of evidence-informed practice, programs, policies?
• How do prevailing gender norms or gender roles serve as barriers or enablers to the uptake of evidence-informed practices, programs, policies?
• When and how should implementation interventions be tailored to the sex, gender and diversity of the target audience?
• Do cognitive and emotional learning strategies differ across sexes or genders, and if so how?
• When and how should the wording or messaging included in the implementation intervention be tailored differently across sex, gender and other identity characteristics?
• How does the implementation intervention increase or decrease gender inequities in socio-economic status, cultural or ethnic groups, and political contexts?
• Does the implementation intervention work differently for sub-groups of men, women and gender-diverse people, and if so, how?
• When using participatory/collaborative or integrated knowledge translation research approaches, does the sex and gender of the researchers and knowledge users matter, and if so, how?
• How do gender relations as a function of dyads or interpersonal dynamics within an organization, community, workplace or institution influence the outcome of the intervention?
• Are there unintended impacts of implementation that exacerbate or diminish sex, gender or other diversity-related inequities?

Another way to study issues of sex and gender in IRP resides in the realist approach which attempts to answer the question, “What works, for whom, in what circumstances, and why?” [47] This is accomplished through the identification and examination of underlying generative mechanisms or program theories associated with the implementation intervention or program, the conditions or contexts under which the mechanisms operate, and the pattern of outcomes produced. Realist evaluators may wish to examine sex and gender through the lens of this Context-Mechanism-Outcomes configuration for the evaluation of new initiatives, programs and scale-up [71, 72].

Through this lens, context can be defined as the particular sub-groups for whom the outcomes were successful, the gender relations between the stakeholders, the sex and/or gender of the individuals who implemented the intervention, and the institutional, socio-economic, cultural and political conditions. Mechanism refers to the explanation of how a particular program’s resources work to change the reasoning and responses of participants to bring about the adoption of the clinical intervention or program that results in both intended and unintended outcomes. Outcomes are the impacts of the intervention. Some questions of how sex and gender considerations can align with the Context-Mechanism-Outcomes configuration are: How do gender roles, gender identity, gender relations, and institutionalized gender influence the way in which an implementation strategy works, for whom, under what circumstances and why? Or, how do program theories operate/work within and across sexes, genders and other diversity characteristics, in what circumstances and why? Finally, research results should be disaggregated and reported by sex or gender groups [11]. It is important to report whether there are similar effects or differences.

When critically appraising the publications of implementation research, reviewers should increasingly ask whether the reports consider sex and gender during a study’s life cycle, and if so, how? Table 3 provides a beginning list of questions that can be asked of implementation research to help the reader assess whether sex and gender have been adequately considered, and the extent to which this may have influenced the study findings and conclusions.

**Table 3:** Questions to ask when appraising an implementation research or practice initiative for inclusion of sex and gender considerations

• Has the systematic review of the effectiveness of implementation interventions considered evidence related to sex and gender?
• Has the literature review and analysis of the know-do-gap considered gender roles, gender identity, gender relations, institutionalized gender?
• Does the monitoring and evaluation plan for the intervention collect data on sex, gender and diverse factors, and include a strategy for assessing and mitigating inequitable outcomes?
• Has the assessment of barriers and facilitators of the use of evidence-informed practices, programs, policies considered gender roles, gender identity, gender relations, institutionalized gender?
• Has the process by which local or targeted adaptation of the evidence-informed practices, programs, policies considered cultural contexts of gender roles, gender identity, gender relations, institutionalized gender?
• Has the implementation intervention been tailored to address sex, gender or other identity or diversity-related characteristics identified in the barriers assessment?
• Has knowledge use (uptake of the practice, program, policy) been reported by sex, gender, and other population characteristics such as age, socioeconomic status etc?
• Have health outcomes (impact of adopting the practice, program, policy) been reported by sex, gender, and other population characteristics?
• Has the impact of unintended consequences of implementation been reported by sex, gender, and other population characteristics?

## Conclusion

This paper argues that sex and gender should always be considered in implementation research. Considering sex and gender should be an essential component of IRP. Failing to integrate sex and gender may neglect an important determinant of knowledge use, reducing the effectiveness of implementation interventions, inadvertently reinforcing sex neutral claims and negative gender stereotypes, and possibly creating or increasing gender and health inequities in care and health outcomes. Only by consistently investigating sex and gender in a critical and reflective manner that addresses underlying gender inequities, will the field of IRP reach its full potential for meeting the requirements of scientific rigour, excellence and maximal impact.

## Additional file

Additional file 1: Appendix 1. List of the top implementation science papers published in 2015. (DOCX 19 kb)

## Abbreviations

EPOC: Effective Practice and Organisation of Care
IRP: Research and practice
