# Supplementary material for: Why sex and gender matter in implementation research
Source: BMC Med Res Methodol. 2016 Oct 27;16:145. doi: 10.1186/s12874-016-0247-7 (PMC5084413; doi:10.1186/s12874-016-0247-7)
Supplement: Additional file 1: — Appendix 1. List of the top implementation science papers published in 2015. (DOCX 19 kb) [file 12874_2016_247_MOESM1_ESM.docx]

| **Appendix 1 : 2015 Top Articles in Implementation Science** |
| --- |

| 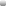 | [The Society for Implementation Research Collaboration Instrument Review Project: A methodology to promote rigorous evaluation](http://www.implementationscience.com/content/10/1/2) |
| --- | --- |
| 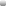 | [Co-production in practice: how people with assisted living needs can help design and evolve technologies and services](http://www.implementationscience.com/content/10/1/75) |
| 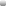 | [What's in a mechanism? Development of a key concept in realist evaluation](http://www.implementationscience.com/content/10/1/49) |
| 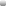 | [A refined compilation of implementation strategies: results from the Expert Recommendations for Implementing Change (ERIC) project](http://www.implementationscience.com/content/10/1/21) |
| 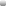 | [Learning Evaluation: blending quality improvement and implementation research methods to study healthcare innovations](http://www.implementationscience.com/content/10/1/31) |
| 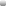 | [Developing a checklist for guideline implementation planning: review and synthesis of guideline development and implementation advice](http://www.implementationscience.com/content/10/1/19) |
| 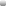 | [Leadership and organizational change for implementation (LOCI): a randomized mixed method pilot study of a leadership and organization development intervention for evidence-based practice implementation](http://www.implementationscience.com/content/10/1/11) |
| 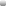 | [What strategies are used to build practitioners' capacity to implement community-based interventions and are they effective?: a systematic review](http://www.implementationscience.com/content/10/1/80) |
| 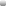 | [Making sense of implementation theories, models and frameworks](http://www.implementationscience.com/content/10/1/53) |
| 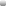 | [Developing standards for reporting implementation studies of complex interventions (StaRI): a systematic review and e-Delphi](http://www.implementationscience.com/content/10/1/42) |
